# Differential impact of the COVID-19 pandemic on primary care utilization related to common mental disorders in four European countries: A retrospective observational study

**DOI:** 10.3389/fpsyt.2022.1045325

**Published:** 2023-01-09

**Authors:** Pär Flodin, Alma Sörberg Wallin, Barbara Tarantino, Paola Cerchiello, Karolína Mladá, Marie Kuklová, Lucie Kondrátová, Enea Parimbelli, Walter Osika, Anna-Clara Hollander, Christina Dalman

**Affiliations:** ^1^Department of Global Public Health, Karolinska Institutet, Stockholm, Sweden; ^2^Department of Brain and Behavioral Sciences, University of Pavia, Pavia, Italy; ^3^Department of Economics and Management, University of Pavia, Pavia, Italy; ^4^Department of Public Mental Health, National Institute of Mental Health, Klecany, Czechia; ^5^Department of Psychiatry, Faculty of Medicine, University Hospital in Pilsen, Charles University, Prague, Czechia; ^6^Department of Demography and Geodemography, Faculty of Science, Charles University, Prague, Czechia; ^7^Department of Electrical, Computer and Biomedical Engineering, University of Pavia, Pavia, Italy; ^8^Telfer School of Management, University of Ottawa, Ottawa, ON, Canada; ^9^Department of Clinical Neuroscience, Centre for Psychiatry Research, Karolinska Institutet, Stockholm, Sweden; ^10^Department of Neurobiology, Care Sciences and Society, Center for Social Sustainability, Karolinska Institutet, Stockholm, Sweden; ^11^Center for Epidemiology and Community Medicine, Stockholm, Sweden

**Keywords:** COVID-19, primary healthcare, common mental disorder, containment measures, time series analysis

## Abstract

**Background:**

The COVID-19 pandemic is commonly believed to have increased common mental disorders (CMD, i.e., depression and anxiety), either directly due to COVID-19 contractions (death of near ones or residual conditions), or indirectly by increasing stress, economic uncertainty, and disruptions in daily life resulting from containment measure. Whereas studies reporting on initial changes in self-reported data frequently have reported increases in CMD, pandemic related changes in CMD related to primary care utilization are less well known. Analyzing time series of routinely and continuously sampled primary healthcare data from Sweden, Norway, Netherlands, and Latvia, we aimed to characterize the impact of the pandemic on CMD recorded prevalence in primary care. Furthermore, by relating these changes to country specific time-trajectories of two classes of containment measures, we evaluated the differential impact of containment strategies on CMD rates. Specifically, we wanted to test whether school restrictions would preferentially affect age groups corresponding to those of school children or their parents.

**Methods:**

For the four investigated countries, we collected time-series of monthly counts of unique CMD patients in primary healthcare from the year 2015 (or 2017) until 2021. Using pre-pandemic timepoints to train seasonal Auto Regressive Integrated Moving Average (ARIMA) models, we predicted healthcare utilization during the pandemic. Discrepancies between observed and expected time series were quantified to infer pandemic related changes. To evaluate the effects of COVID-19 measures on CMD related primary care utilization, the predicted time series were related to country specific time series of levels of social distancing and school restrictions.

**Results:**

In all countries except Latvia there was an initial (April 2020) decrease in CMD care prevalence, where largest drops were found in Sweden (Prevalence Ratio, PR = 0.85; 95% CI 0.81–0.90), followed by Netherlands (0.86; 95% CI 0.76–1.02) and Norway (0.90; 95% CI 0.83–0.98). Latvia on the other hand experienced increased rates (1.25; 95% CI 1.08–1.49). Whereas PRs in Norway and Netherlands normalized during the latter half of 2020, PRs stayed low in Sweden and elevated in Latvia. The overall changes in PR during the pandemic year 2020 was significantly changed only for Sweden (0.91; 95% CI 0.90–0.93) and Latvia (1.20; 95% CI 1.14–1.26). Overall, the relationship between containment measures and CMD care prevalence were weak and non-significant. In particular, we could not observe any relationship of school restriction to CMD care prevalence for the age groups best corresponding to school children or their parents.

**Conclusion:**

Common mental disorders prevalence in primary care decreased during the initial phase of the COVID-19 pandemic in all countries except from Latvia, but normalized in Norway and Netherlands by the latter half of 2020. The onset of the pandemic and the containment strategies were highly correlated within each country, limiting strong conclusions on whether restriction policy had any effects on mental health. Specifically, we found no evidence of associations between school restrictions and CMD care prevalence. Overall, current results lend no support to the common belief that the pandemic severely impacted the mental health of the general population as indicated by healthcare utilization, apart from in Latvia. However, since healthcare utilization is affected by multiple factors in addition to actual need, future studies should combine complementary types of data to better understand the mental health impacts of the pandemic.

## 1. Introduction

Since the onset of the COVID-19 pandemic there has been a growing concern of the consequences the COVID-19 pandemic and the associated containment measures will have on population mental health (1, 2). Prior to the pandemic, the most common mental disorders were anxiety, with a global prevalence of 3.8% (4.9% in Europe), followed by depression (3.6% globally, and 4.2% in Europe) (3). In Europe, anxiety disorders contribute 1.4% (1.0–1.8%) to the total disease burden (Disability Adjusted Life Years, DALYs) and depression by 2.2% (1.6–2.9%) (3). Taken together, anxiety and depression, here referred to as common mental disorders (CMD), constitutes 62% percent of the total burden of mental disorders in Europe.

The COVID-19 pandemic and associated containment measures increased the exposure to psychological stressors, including loneliness, boredom, economical stress, and domestic violence (4, 5). At the same time, certain population groups might have experienced relaxed work schedules, reduced time commuting, and less stress in daily life, protecting them from adverse mental health effects (6, 7). Hence, the psychological consequences of the pandemic likely differ across demographic groups, timescales, and countries. The varying degrees to which governments imposed policies aimed to control the pandemic provides an opportunity to evaluate which policies have the biggest impacts on mental health.

Exploring the indirect effects of the pandemic and the pertaining regulations is important for two reasons. Firstly, a better view of the need for healthcare in the wake of a pandemic would facilitate public health planning and help optimize healthcare spendings. Secondly, knowing which measures have the greatest adverse health consequences could inform how future pandemics should be handled.

Tracking changes in population mental health in the wake of societal crises such as the COVID-19 pandemic are conventionally accomplished using self-reports, suicide rates, psychotropic prescription rates or healthcare utilization. Most studies investigating the impact of the pandemic relies on the former. However, time trends of health utilization data provide important complementary information to the more commonly employed survey data. Firstly, the availability of routinely collected healthcare data is high, and since continuously recorded it typically allows for better pre-pandemic baseline comparisons with higher time resolution as compared to survey studies that relies on only one or a few baseline measurements. Secondly, healthcare utilization records are based on clinically validated diagnoses determined by clinicians, whereas survey studies, although when employing validated psychometric assessment scales, rely on less valid self-reported data that commonly overestimate the rates of mental illness (8). Thirdly, healthcare utilization data typically provides a more complete coverage of the study population than what is feasible using survey data.

Among studies that previously investigated COVID-19 related changes in healthcare utilization pertaining to CMD, only few use primary care data. Such data are exceptionally valuable for detecting changes in public mental health, given that the majority (around 60–90%) of European (Swedish and British) patients that seek help for CMDs only encounter primary care centers (rather than specialized out- or inpatient care) (9, 10).

Previous studies of primary care recorded CMD changes typically report a sharp decrease in the number of patients during the first months of the pandemic, followed by a normalization during the latter half of 2020. For instance, Carr et al. (11) reported dramatic decreases in CMD incidence during April 2020 in the UK, although already by June 2020 depression levels were normalized, and anxiety levels nearly normalized. However, Carr et al. (11) only report on data until September 2020, preventing any conclusions on the long-term effects of the enduring pandemic. Initial decreases in depressive disorders have also been reported in Germany (12), Norway (13), Spain (14), where in the latter case levels stayed low until early 2021.

However, studies from Denmark (15) and Netherlands (16) have reported unchanged, or in the latter case, slightly increased CMD levels. In both studies, the authors attribute the relatively constant CMD levels to efficient transfers of the mode of care delivery, from face-to-face to virtual consultations.

Taken together, previous studies report a varying degree of changes in the number of CMD patients in primary care. In most countries, the onset of the pandemic was followed by an initial decrease in CMD cases, whereas a few countries experienced immediate increases that typically were followed by decreases. Although most of the above studies reported on data until the end of 2020 (and hence blind to long term effects), the majority reported normalized CMD levels during the latter part of 2020. Unfortunately, several of these studies looked at rather limited periods of pre-pandemic data or used analytical approaches that lacked control of long-term trends in healthcare utilization, which thus could render misleading conclusions.

In this study, we investigated changes in the number of unique individuals seeking care for CMD during approximately the first year of the COVID-19 pandemic compared to the previous 3–5 years. Although data availability and the organization of healthcare systems varied across countries, we aimed to collect as comparable measures of care utilization as possible. Previous studies suggest that among all mental disorders, CMD would belong to the most affected (11, 16, 17). Since, at least in Sweden and the UK, most individuals seeking care for CMD meet general practitioners rather than specialists [Flodin et al., n.d., (9, 10)], CMD counts in primary care would likely be among the most sensitive measures for changes in population mental health. To our knowledge, this is the first study that investigates the effect of the pandemic on CMD related primary healthcare utilization in multiple countries. Furthermore, this is the first attempt to evaluate the variation of intensities of several types of restrictions taken by governments in multiple countries, in order to understand which restrictions that have the greatest effects on mental health.

## 2. Materials and methods

### 2.1. Collection of mental health data

Data on primary healthcare utilization were obtained from seven national or regional databases of routinely and continuously, near real time records of primary care visits. European database owners were identified through literature searches on PubMed (Supplementary Table 1) and Google scholar, requests in collegial networks of European public health professionals, internet searches, and using snowballing strategies. Given that our scope was to investigate the effects of the pandemic containment measures in an European context, searches were restricted to European countries. Data availability was generally scarce, and due to the ongoing pandemic the register owners frequently reported having longer processing time than usual. Eventually, we obtained tailored datasets from four countries (and seven independent databases).

#### 2.1.1. Norway

We received national data from the Norwegian Control and Payment of Health Reimbursements Database (the KHUR-register) owned by the Norwegian Directorate of Health. Data spanned the period between January 2017 to June 2021. Norwegian control and payment of health reimbursements database (KHUR) provided virtually full national coverage of publicly funded primary care visits in Norway (18), i.e., 5.40 million inhabitants (December 2020). The data set comprised 130.01 million primary care encounters (regardless of diagnosis), rendering a monthly average of 2.41 million patient visits.

#### 2.1.2. Latvia

Data was received from the Latvian Center for Disease Prevention and Control (19). The database contained primary healthcare utilization records pertaining to the full Latvian population, i.e., 1.90 million in December 2020. The Latvian data set comprised 27.16 million encounters, or 0.38 million encounters per month.

#### 2.1.3. Sweden

We obtained primary care data from the four counties (Stockholm County, Västra Götaland County, Skåne County, and Östergötland County), together covering 6.03 million or approximately 59% of the Swedish population (December 2020) (20–23). Coverage rates are unknown but should contain virtually all publicly financed primary healthcare. The full data set comprised 197.96 million encounters, or 2.64 million monthly encounters.

#### 2.1.4. Netherlands

Dutch primary care data was obtained from the Nivel Primary Care Database (founded by Netherlands Ministry of Public Health, Welfare and Sports) covering a population of 17.40 million (December 2020) albeit with an undetermined coverage rate (24). The Dutch data set comprised 410.67 million encounters, or 5.70 million encounters each month.

### 2.2. Outcome

Anxiety disorders and depression were defined according to the nomenclature used by the Global Burden of Disease project (GBD) [defining International Classification of Diseases (ICD10)–and International Classification of Primary Care (ICPC) codes are listed in Supplementary Table 2]. From all registers, we requested aggregated number of unique individuals diagnosed with anxiety or depression, stratified by age group, sex, region, and months of visit (see Section “Analysis”).

The Swedish Ethical Review Authority, Uppsala, deemed that no ethical permit was required for the study, as it only reports on aggregated and thus anonymized, already collected register data (reference 2021-01501).

### 2.3. Containment measure indices

Indices of containment measures were provided by Goldszmidt et al. (25)^1^ and constituted six indices that measured intensity of government response to tackle the pandemic. These were *social distancing*, *school restrictions*, *businesses restrictions*, *health monitoring*, *health resources*, and *mask wearing*. Indices consisted of country specific, daily estimates from 1 January 2020 until October 2021 for the investigated four (and additional more than 180) countries. The indices were created by combining two of the most comprehensive COVID-19 datasets: the Corona Net COVID-19 Government Response Event Dataset, and the Oxford COVID-19 Government Response Tracker, and are described in greater detail elsewhere (25). Due to the high degree of collinearity among the six available indices and in order to reduce the problem of multiple comparisons, we selected only the two most plausible to be relevant for population mental health, namely social distancing and schools’ restrictions. The social distancing index captures restrictions on individual mobility, such as lockdowns, travel bans etc. The index on schools’ restrictions captured provisions of education, such as closure of secondary schools, mandated distance educations, postponements of school exams etc.

### 2.4. Analysis

Data was structured in time series format with monthly counts of the number of unique individuals visiting primary care and who got registered with anxiety disorders or depression as main diagnoses. Hence, for each psychiatric disease, an individual was counted only once per month, although the same individual could be counted at two different calendar months, or once for depression and once for anxiety during the same months. The CMD variable was the sum of the anxiety and the depression counts. All time series were normalized by population denominator corresponding to the population coverage of each register and time point, rendering the outcome unit “number of unique individuals registered with CMD in primary care per 100 000 person-months” (subsequently referred to as “CMD per 100,000 pms”).

For each region, months and demographic group, expected prevalence rates were estimated using models trained on the pre-pandemic data, typically spanning from January 2015 (or in the case of Norway, from January 2017) until December 2019. For time series predictions, we used seasonal ARIMA (Auto Regressive Integrated Moving Average) models. Briefly, ARIMA predicts future time series based on a historical time series. ARIMA models serial correlation (by the Auto-Regressive “AR” term, and the Moving Average “MA” term), non-stationarity (by differencing or the “I” term) and seasonal trends. Hence seasonal ARIMA accounts for both long term trends (i.e., yearly changes), seasonality (monthly changes), and changes of these over time. A 12 months seasonality was imposed on the otherwise automatically derived ARIMA model parameters. The parameters were selected by stepwise comparisons of goodness-of-fit as quantified by the Akaike information criterion (AIC), as implemented in the python library *pmdarima 2.0.1*.

Predictions were subsequently compared to actual, observed prevalence rates, and for each month, a prevalence ratio (PR) was obtained by dividing observed by expected prevalence rates. A total of 95% confidence intervals were calculated using bootstrap resampling with replacement using 500 iterations. Thus, for each country, demographic group, and time period, we got PR indicating the deviation between observed and expected CMD prevalence in the primary care.

Then we tested for the relationship between containment measures and the deviations in CMD care prevalence. The indices were retrieved from https://github.com/CoronaNetDataScience/corona_index. The raw indices were transformed by first shifting them to start at 0 when the pandemic started. Then they were scaled as to span the range 0–100, where 100 denoted the maximum value reached for each index in any of the four countries.

For each country and each demographic group, we specified two separate models consisting of a policy index pertaining to either school restrictions or to social distancing. Additionally, we included a binary variable of no interest pertaining to the onset of the pandemic (consisting of 0 s in time points prior to pandemic, and 1′s during the pandemic, with onset set at March 2020). The dummy variable was included to control for general pandemic effects unrelated to the investigated policy restrictions at hand, such as fear of infections, economic stress, or official recommendations to avoid healthcare services unless necessary. All regressors (i.e., school restrictions, social distancing, and the binary pandemic regressor) within each country were highly correlated (*r* > 0.83, *p* < 0.001).

Since we had no strong *a priori* reasons to estimate linear relationships between levels of containment measures and CMD care prevalence, we performed a sensitivity analysis testing for non-linear (polynomial of the second order) effects as reported in Supplementary Table 3. All data analyses were performed using Python 3.6, and the code is available at https://github.com/parflo/pandemic_CMD_in_europe/.

## 3. Results

### 3.1. Baseline prevalence rates

**FIGURE 1:**
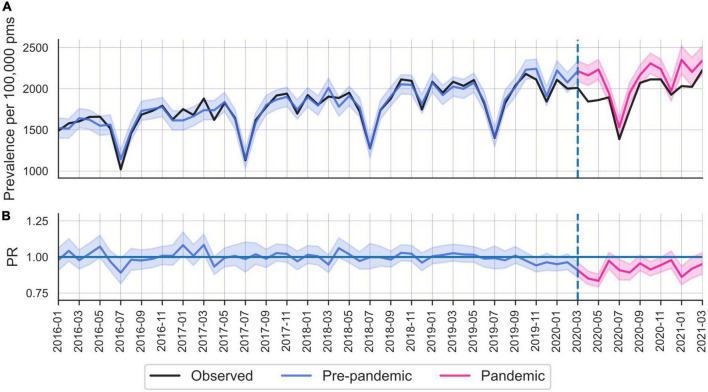
**(A)** Number of unique individuals seeking care for depression and/or anxiety. Monthly counts in Sweden between January 2016 and March 2021, all ages and both sexes. Black = actual number; blue = fitted prevalence rates (with 95% confidence interval), pink = predicted prevalence rates (95% CI). **(B)** Prevalence ratios (i.e., observed over expected) (95% CI). Vertical striped lines indicate onset of pandemic (March 2020) (the first year of this and subsequently presented time series provides unreliable model fits and are omitted for display purposes).

**FIGURE 2:**
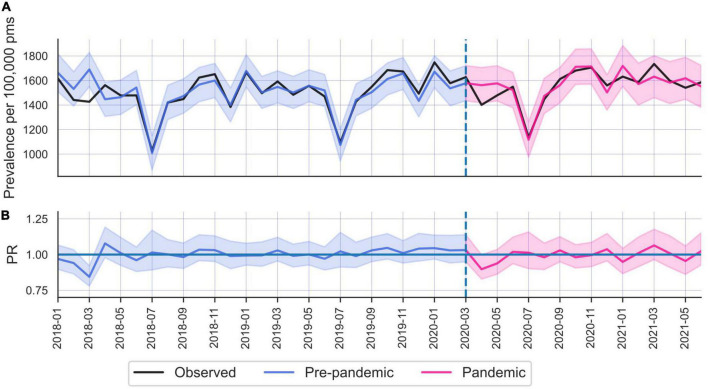
**(A)** Common mental disorders (CMD) care prevalence in Norway, during January 2018 until May 2021. **(B)** Prevalence ratios.

**FIGURE 3:**
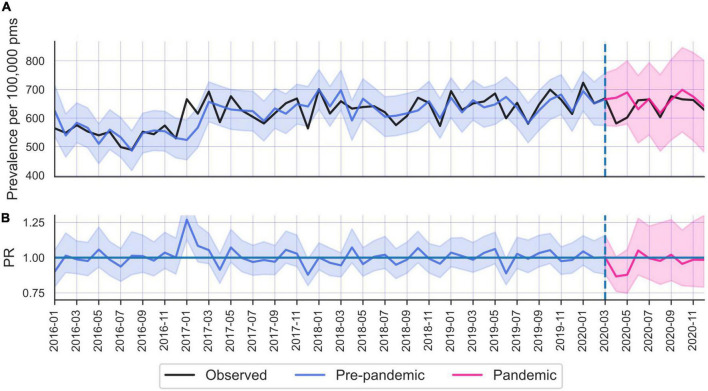
**(A)** Common mental disorders (CMD) care prevalence in the Netherlands. **(B)** Prevalence ratios.

**FIGURE 4:**
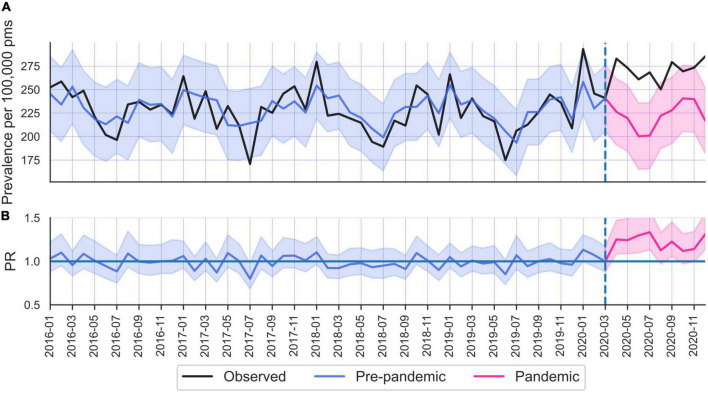
**(A)** Common mental disorders (CMD) care prevalence in Latvia. **(B)** Prevalence ratios.

Baseline CMD care prevalence (i.e., average monthly counts during the 12 months period prior to the pandemic onset in March 2020) varied widely across the countries, with highest levels in Sweden (1,963 per 100,000 person-months), followed by Norway (1,529 per 100,000 person-months), Netherlands (653 per 100,000 person-months), and lastly Latvia (227 per 100,000 person-months). Also, the baseline distribution of anxiety vs. depression cases varied greatly (Table 1). However, within each country, both disorders displayed similar changes over time (Supplementary Figures 1, 2). Strong seasonal patterns were observed primarily for the Swedish and Norwegian population, and less so for the Dutch and Latvian time series. Also, long term increases were most prominent for Sweden, followed by Norway (Figures 1–4).

**TABLE 1:** Baseline prevalence in primary care for common mental disorders, anxiety, any psychiatric conditions, and any diagnosis, respectively.

	CMD (per 100,000 pms)	Anxiety (per 100,000 pms) (% of CMD)	Psychiatric diagnosis (per 100,000 pms)	Any diagnose (per 100,000 pms)
Sweden	1,963	1,409 (72%)	2,480	21,659
Norway	1,529	789 (48%)	3,646	27,062
Netherlands	653	281 (43%)	1,423	21,427
Latvia	227	190 (84%)	356	17,384

pms, person-months.

Spurred by the variation in CMD baseline care prevalence (e.g., almost seven times higher rates in Sweden compared to Latvia), we further scrutinized the coverage rates of each register. Since we did not have access to formal estimations of coverage rates, we compared the countries with regard to two indicator variables: monthly number of unique individuals utilizing primary healthcare receiving *any* diagnosis, and the number of unique individuals receiving *any psychiatric* diagnosis (Table 1 and Supplementary Figures 3, 4).

### 3.2. Changes in prevalence during the pandemic

#### 3.2.1. Sweden

The Swedish population (all ages and both sexes combined) displayed significantly reduced CMD care prevalence from March 2020 until December 2020 (PR = 0.91; 95% CI 0.90–0.93), (Table 2). There was a clear age gradient in CMD changes, where the youngest age group displayed an increased prevalence ratio (PR = 1.08; 95% CI 1.04–1.11), while the pandemic associated decrease in CMD care prevalence became more pronounced with older age groups. Further investigations into sex differences revealed that the age dependent pattern of CMD change was driven by females, whereas males did not display overall trends of greater reductions among the older age groups (Table 2).

**TABLE 2:** Prevalence ratios (observed divided by expected) of common mental disorders (CMD) during the pandemic (Mars 2020-December 2020) in Sweden, Norway, Netherlands, and Latvia during the first year of the COVID-19 pandemic (March 2020-December 2020).

		Sweden	Norway	Netherlands	Latvia
Both sexes	All ages	0.91 (0.90, 0.93)*	0.99 (0.96, 1.02)	0.97 (0.91, 1.03)	1.20 (1.14, 1.26)*
	0–14	1.08 (1.04, 1.11)*	0.91 (0.87, 0.96)*	0.94 (0.83, 1.07)	0.83 (0.76, 0.90)*
	15–29	0.97 (0.95, 0.99)*	0.96 (0.93, 1.00)	0.92 (0.87, 0.99)*	0.98 (0.94, 1.03)
	30–44	0.93 (0.91, 0.95)*	1.00 (0.97, 1.03)	0.98 (0.92, 1.07)	1.18 (1.12, 1.24)*
	45–59	0.90 (0.88, 0.91)*	1.02 (0.99, 1.05)	0.99 (0.95, 1.04)	1.30 (1.25, 1.34)*
	60–74	0.87 (0.85, 0.89)*	1.08 (1.05, 1.11)*	1.00 (0.95, 1.05)	1.43 (1.35, 1.52)*
	75–89	0.90 (0.86, 0.94)*	1.06 (1.02, 1.09)*	1.03 (0.99, 1.08)^1^	1.80 (1.66, 1.97)*
	90+	0.88 (0.83, 0.93)*	1.13 (1.07, 1.21)*		2.14 (1.72, 2.76)*
Females	All ages	0.91 (0.89, 0.93)*	0.99 (0.96, 1.02)	0.97 (0.91, 1.04)	1.22 (1.18, 1.27)
	0–14	1.13 (1.09, 1.17)*	0.93 (0.88, 0.99)*	0.82 (0.72, 0.94)*	0.81 (0.73, 0.93)*
	15–29	0.98 (0.96, 0.99)*	0.96 (0.93, 1.00)	0.91 (0.86, 0.96)*	0.98 (0.93, 1.05)
	30–44	0.92 (0.90, 0.93)*	1.00 (0.97, 1.03)	0.99 (0.91, 1.07)	1.18 (1.12, 1.24)*
	45–59	0.89 (0.87, 0.91)*	0.99 (0.96, 1.03)	0.96 (0.92, 1.01)	1.28 (1.22, 1.34)*
	60–74	0.86 (0.84, 0.88)*	1.02 (0.99, 1.04)	1.01 (0.95, 1.08)	1.44 (1.36, 1.54)*
	75–89	0.88 (0.85, 0.92)*	1.06 (1.02, 1.10)	1.06 (1.02, 1.11)^1^	1.82 (1.65, 2.02)*
	90+	0.87 (0.82, 0.91)*	1.12 (1.06, 1.18)		1.88 (1.55, 2.45)*
Males	All ages	0.93 (0.91, 0.95)*	0.99 (0.96, 1.02)	0.96 (0.91, 1.03)	1.16 (1.11, 1.22)*
	0–14	0.99 (0.95, 1.03)	0.88 (0.83, 0.93)*	1.04 (0.93, 1.19)	0.84 (0.75, 0.93)*
	15–29	0.97 (0.94, 0.99)*	0.97 (0.94, 1.00)	0.96 (0.90, 1.03)	0.99 (0.93, 1.07)
	30–44	0.95 (0.93, 0.97)*	1.01 (0.98, 1.05)	0.98 (0.91, 1.07)	1.20 (1.13, 1.30)*
	45–59	0.91 (0.89, 0.93)*	1.02 (0.99, 1.05)	0.96 (0.92, 1.01)	1.28 (1.20, 1.37)*
	60–74	0.88 (0.86, 0.90)*	1.05 (1.03, 1.08)*	0.99 (0.96, 1.04)	1.40 (1.31, 1.50)*
	75–89	0.96 (0.91, 1.01)	1.12 (1.08, 1.16)*	1.02 (0.95, 1.08)^1^	1.98 (1.75, 2.29)*
	90+	0.98 (0.93, 1.04)	1.20 (1.01, 1.44)*		1.40 (1.06, 2.10)*

*PRs significantly different from 1, i.e., statistically significant deviation from expected prevalence numbers.

^1^Due to limited age resolution in the Dutch data, the oldest age group presented for the Netherlands are 75+ years. Results are stratified by age and sex.

Analysis of CMD changes on a monthly basis revealed the largest decreases occurred in April and May 2020 (in April PR 0.85; 95% CI 0.81–0.90). Levels returned to near normal in June 2020, to again drop below expected levels during the fall in 2020, albeit not as dramatically (Figure 1).

#### 3.2.2. Norway

Norway did not display an overall change in CMD care prevalence among the total population during the first year (i.e., 10 months) of the pandemic (PR 0.99; 95% CI 0.96–1.02). Interestingly, compared to Sweden, Norway displayed an inverted pattern of age dependent CMD change, where younger age groups decreased more than older. Furthermore, this age difference was more pronounced among males than females (Table 2). Similar to Sweden, the largest CMD drop occurred during spring 2020 (e.g., in April PR 0.90; 95% CI 0.83–0.97) and was normalized by June 2020 (Figure 2).

#### 3.2.3. Netherlands

The Netherlands presented a similar pattern of change in CMD (Figure 3) as Norway, where no significant changes occurred for the population at large. However, young adults (particularly females) displayed the largest decrease in CMD rates. Women 0–14 years old displayed a 18% drop in prevalence (PR 0.82; 95% CI 0.72–0.94), whereas females 75 + showed a 6% increase (PR 1.06; 95% CI 1.02–1.11) (Table 2).

#### 3.2.4. Latvia

Latvia was the only country where CMD increased in the total population (PR 1.29; 95% CI 1.14–1.26) during April to December 2020 (Table 2). Although the increase was largest among women (PR 1.22; 95% CI 1.18–1.27) compared to men (1.16; 95% CI 1.11–1.22), both sexes displayed CMD increase in older age groups. The elevated levels occurred in April 2020 and remained more or less elevated throughout 2020 (Figure 4).

### 3.3. Increased virtualization of primary care

From Sweden and Norway, we had data on the degree of virtualization (i.e., the number of primary care consultations that took place either online, *via* mobile app or on telephone). Both countries underwent a sudden and dramatic virtualization of primary mental healthcare. In Sweden, during the 12 months prior to pandemic, 26.5% of all CMD diagnoses were recorded from tele consultations, compared to 44.6% during the first 12 month of the pandemic (April 2020 until March 2021), (i.e., 73.6% increase), (see Supplementary Figures 5, 6). Correspondingly, Norway experienced a shift from 18.0% teleconsultation to 39.2%, (i.e., 117% increase).

### 3.4. Effects of containment measures

The relationship between CMD care prevalence and containment measures (i.e., school restrictions and social distancing) were generally weak (Table 3). None of the investigated policy indices displayed significant associations (Bonferroni corrected *p*-value at an alpha level of 0.05/32 = 0.0016). However, for the populations at large, there was a tendency (defined as alpha levels of 0.05 uncorrected for multiple comparisons) of a negative association between school restrictions and decreased CMD care prevalence in the Netherlands such that one point increase in school restrictions decreased CMD care prevalence (beta = −2.7 × 10^–5^, *p* = 0.024), or 459 fewer individuals each month in whole country. Similarly, in Latvia there was a tendency of negative associations between school restrictions and CMD (beta = −9.2 × 10^–6^, *p* = 0.022, corresponding to less than one person decrease each month). Contrary to our *a priori* hypothesis, we did not observe any selective effect of school restrictions in the age groups that best corresponded to school children (0–14 years) or their parents (30–59 years) in any country.

**TABLE 3:** Policy predicted common mental disorders (CMD) care prevalence by age groups in all countries.

Region	Measure	All ages, beta (*p*)	0–14 years, beta (*p*)	30–59 years, beta (*p*)	75+ years, beta (*p*)
Sweden	SD	6.6 × 10^–5^ (0.34)	1.2 × 10^–5^ (0.0030)*	6.0 × 10^–5^ (0.65)	7.3 × 10^–5^ (0.21)
	SR	1.0 × 10^–4^ (0.71)	1.2 × 10^–6^ (0.95)	1.0 × 10^–4^ (0.98)	2.0 × 10^–4^ (0.60)
Norway	SD	2.4 × 10^–5^ (0.81)	9.9 × 10^–6^ (0.037)*	1.4 × 10^–5^ (0.90)	4.5 × 10^–5^ (0.33)
	SR	3.1 × 10^–5^ (0.71)	−7.4 × 10^–6^ (0.31)	9.0 × 10^–5^ (0.55)	1.4 × 10^–6^ (0.98)
Netherlands	SD	−6.0 × 10^–6^ (0.27)	−2.0 × 10^–6^ (0.25)	−6.0 × 10^–6^ (0.48)	−4.2 × 10^–6^ (0.21)
	SR	−2.7 × 10^–5^ (0.024)*	−2.2 × 10^–6^ (0.48)	−3.5 × 10^–5^ (0.057)	−3.0 × 10^–6^ (0.80)
Latvia	SD	−9.2 × 10^–6^ (0.022)*	−1.9 × 10^–6^ (0.39)	−9.0 × 10^–5^ (0.090)	−5.2 × 10^–6^ (0.024)*
	SR	−7.2 × 10^–6^ (0.20)	7.7 × 10^–6^ (0.53)	−8.3 × 10^–6^ (0.17)	−3.7 × 10^–6^ (0.63)

SD, social distancing; SR, school restrictions.

*Denotes associations that are significant at *p* < 0.05, uncorrected.

Similarly, in a follow up analysis testing for non-linear (quadratic) relationships between policy and CMD, no significant associations were observed (Supplementary Table 3).

## 4. Discussion

In the current multinational, retrospective observational study we observed several notable pandemic associated changes in CMD rates. During the initial phase of the COVID-19 pandemic (April- May 2020) the prevalence of primary care-recorded CMD dropped in Sweden, Norway, and the Netherlands, but increased in Latvia. In Norway and the Netherlands, the prevalence returned to baseline during the summer 2020 and then remained close to expected levels until the end of the study period (March 2021). Meanwhile, Sweden and Latvia maintained decreased and increased CMD care prevalence, respectively.

Overall, the PR during the pandemic (March to December 2020) dropped in Sweden (by 9%), remained roughly as expected in Norway and the Netherlands, but increased in Latvia (by 20%). Interestingly, while Sweden experienced elevated prevalence rates among the youngest age groups (particularly among females) but decreasing with older age, the reversed age pattern was observed in the other three countries: In Norway, the Netherlands, and Latvia prevalence rates increased with age.

In Sweden and Netherlands (albeit in opposite directions), the age gradient in prevalence ratios were primarily driven by females, whereas in Norway males displayed the largest age differences. Latvia did not display any clear age by sex interactions.

The initial drop in CMD related primary care utilization and a subsequent return to pre-pandemic levels parallels what has been seen elsewhere. During April 2020 in the UK, Carr et al. (11) reported reductions in treated incidence of depression and anxiety by 43% and 47%, respectively, and Williams et al. (26) reported a 50% decrease for the same period. Although (14) reported an initial increase (19%) of anxiety during March 2020 in a Spanish population, this was soon replaced by a large decrease. Furthermore, depression immediately showed a sharp decrease (64%) during the first months (March to June 2020) of the pandemic, and first by early 2021 CMD incidences reached pre lockdown levels.

However, several previous studies report elevated CMD rates, particularly at the second half of 2020. After an initial drop in Norwegian CMD cases (similar to what we found), Hvide and Johnsen (13) reported elevated levels from May holding up through December 2020. This was also the case in a study of a German subpopulation of approximately 3 million individuals. Bohlken et al. (12) compared the CMD incidence in primary care during the last three quarters of 2020 with the average number for similar periods during previous 2 years, and detected an increase by 19% for anxiety during 2020, but no change neither in depression nor in stress disorders. Unfortunately, both these studies suffered similar limitations in analytical strategies where long-term trends were not properly controlled for, which likely contributed to the observed increases and explain why Hvide and Johnsen (13) observed increased CMD in Norway where we did not.

Finally, a Dutch study of both primary and specialized mental care covering data of approximately 2 million inhabitants, albeit from a different register than reported in current study, found near unchanged levels of psychiatric care utilization during 2020. The authors concluded that the constant rates could be explained by increases in both telepsychiatry and video consultations during the first COVID-19 outbreak (16).

Employed measures of CMD rates varied across the previous studies. Bohlken et al. (12) reported on 1 year incidence, i.e., only included patients that had no diagnoses at least 1 year prior to the date they were included. Similarly, Raventós et al. (14) removed all cases with less than 1 year of CMD free registrations to the database. However, the most common measure was number of encounters for a given period of time, regardless of patients previous CMD history (13, 15, 16). None of above-mentioned studies reported healthcare prevalence of monthly number of unique individuals as the current study. Potentially, this could affect the magnitude of reported CMD rates, which for Sweden, Norway, and Netherlands were considerably smaller than the ones previously reported for in UK and Spain.

Differences among the healthcare systems (e.g., care expenditure and accessibility) prevent conclusive country comparisons both of prevalence rates at baseline and over time. However, since the total number of primary care diagnoses were relatively comparable across the investigated countries (Supplementary Figure 3), we argue that the observed low CMD baseline rates in particularly Latvia likely reflect relatively accurate prevalence rather than spuriously induced artifacts due to deficient coverage rates. Estimates of CMD by the Global Burden of Disease study (3) show similar incidence rates among the countries: the number of new cases with depression in the year 2019 was roughly 5,600 (4,800–6,300) per 100,000 in Latvia, and 5,200 (4,700–5,800) per 100,000 in Sweden. Similarly, anxiety incidence was 530 (410–650) per 100,000 in Latvia and 670 (540–800) per 100,000 in Sweden. Although baseline CMD levels in Latvia (227 per 100,000 pms) was much lower than for the other reported countries (e.g., only 12% of CMD levels in Sweden), the rates were on par with (albeit slightly differently defined) incidence rates in Spain [Raventós et al. (14)] and the UK (11). Altogether, current study provides evidence of a larger unmet need in Latvia (27) compared to, e.g., Sweden.

Still, it remains to explain why Latvia displayed prolonged elevated PRs of CMD in contrast to the other countries. We can think of two potential reasons: Firstly, given low baseline prevalence in Latvia, it is likely that the low number of patients reflects a more “distilled” patient group of more severe cases, hence unable to halt care seeking. Secondly, potentially the virtualization of Latvian primary healthcare is more developed than in the other countries, facilitating care seeking in the face of societal look down. Although current study unfortunately lacks data on the degree of virtualization in Latvia, results from Kursīte et al. (28) indicates that remote consolations in primary care were not routinely provided for patients with other non-communicable diseases before the pandemic.

With the onset of the pandemic, health delivery systems worldwide have experienced a shift in the mode of delivery of care, with a dramatic transfer from face-to-face consultation toward teleconsultation. Such changes have been reported both in psychiatric care delivered by GPs (15, 16) and in specialized open care (29, 30). In the US, Zhu et al. (31) found that the declining number of in-person encounters in psychiatric open care was correlated with an increase in telehealth encounters.

In the current study only Swedish and Norwegian data contained information on the degree to which primary healthcare encounters were provided face-to-face or through distance consultations. While Sweden increased CMD distance consultations by 74%, Norway increased by 117%, which contributed to maintained rates in Norway despite falling rates in Sweden during 2020. Speculatively, assuming current Dutch data reflects a similar increase in tele consultations as reported previously from the Netherlands (16), such would provide an explanation why we observed maintained CMD rates in the Netherlands. Further studies should investigate how the degree of virtualization could prevent declines in care seeking behavior among those in need.

With regard to age and sex dependent patterns in PR changes throughout year 2020 (Table 2), we see country specific trends. Previous literature has reported different age and sex dependent pattern of CMD changes. Similar to what we observed in Netherlands, both in UK (11) and Spain (14) the largest declines in CMD PRs during 2020 occurred among young females (and particularly among people living in the most deprived urban areas).

Among the investigated containment measures, none were significantly related to CMD levels. Furthermore, we did not confirm our hypothesis of school restriction induced CMD changes among age groups corresponding to school children or their parents. Regardless of if such association truly exists or not, there are several methodological challenges that reduced the statistical sensitivity to detect any. Firstly, the policy indices and the pandemic dummy variable used to control for non-specific factors were highly correlated, preventing unique variance attributed to the policy indices. The pandemic response involved a wide range of factors affecting mental healthcare utilization. Such confounders are unavoidable in the quasi-naturalistic experiment presented by the pandemic where randomized control designs are infeasible. At best, further variables used for controlling such confounding effects could be employed, although the relative sparsity of data points (approximately 60, of which only 9 deviated from the baseline of 0′s) prevent too complex models and loss of degrees of freedom.

Secondly, assuming a real causal relationship between policy and mental health outcomes would exist, the time dynamics between the two is unknown. Here we attempted to relate monthly counts of CMD cases to the median value of containment measure for that same month. However, it is still an open question what the delay period between the exposure of, for instance school restrictions and the stipulated mental health consequences are. Thirdly, the nature of the relationship between exposure and outcomes are unknown. In absence of other evidence, we parsimoniously tested for linear relationships. Subsequently, in a *post hoc* sensitivity analysis we also tested for quadratic relationships. However, there are likely non-linear relationships and cut off points that better capture the associations, that we again, due to relatively few data points would lack statistical power to properly investigate. Finally, the indices are associated with uncertainty. Although previous studies successfully used current policy indices for studying a variety of pandemic related questions (32, 33), the noise in indices make detection of significant associations more difficult.

Given the high correlation of the policy indices it is not surprising that the impact of each was of similar and negligible magnitude. For a unit increase in social distancing (ranging 0–100), there was a non-significant increase in prevalence numbers by 2.4 individuals per 100,000 person-months in Norway (n.s), and 6.6 individuals in Sweden (n.s). The same change in social distancing was associated with 9.2 fewer cases per 100,000 person-months in Latvia and 6 cases in the Netherlands (n.s). Further research is needed to establish which policy took its greatest toll on mental health, to inform choices of containment strategies in future pandemics, as to maximize the gain in infection control while minimizing the detrimental side effects on mental health.

### 4.1. Explanations for changes in healthcare utilization

Country differences in changes in CMD care prevalence are due to both changes in actual need (caused by changes in exposure to stressors of isolation, economic hardships, but also reduced time commuting, and less stress in daily life etc.) and varying COVID-19 restriction policies. International differences in prevalence changes are furthermore modulated by country specific organization of healthcare systems (e.g., degree of virtualization in the primary healthcare services) and cultural factors influencing help seeking behavior.

Primary care prevalence of any disorder declined by between 15–25% in the first 2 months of the pandemic in all four countries, to later normalize by the second half of 2020 (Supplementary Figure 3). A main driver of the initial decreased CMD care prevalence observed in all countries but Latvia is the general disruption of the healthcare system following lockdown. Primary care partly reallocated to COVID-19 care, leaving little resources to less acute conditions. Furthermore, individuals likely delayed healthcare seeking due to fear of infection and to avoid burdening the health service. Studies explicitly investigating healthcare seeking behavior confirm an increased reluctance to utilize healthcare despite needs of doing so. In a Dutch survey among more than 5,000 individuals 20.2% reported having avoided healthcare despite need (32), similar to what also have been reported in Hungary (33). Hence, there is strong evidence that at least parts of the declines in CMD care prevalence are driven by care avoidance during the pandemic rather than decreased needs, particularly given that early evidence suggested a global increase of CMD prevalence by about 25% (34).

### 4.2. Strengths and limitations

The main strength of the current study is the comparable register data of a sensitive mental health outcome, covering population representative monthly data from more than 764 million primary healthcare encounters (regardless of disease), pertaining to more than 31 million people (December 2020) residing in four countries. The relatively long time series allowed for predictions of prevalence rates taking into account seasonality and long-term trends. To our knowledge this is the largest evaluation of pandemic associated changes in CMD prevalence in primary care to date, and the first study investigating changes on mental healthcare utilization in multiple countries, using the comparable outcome metrics. Furthermore, using country specific indices quantifying the extent of containment measures in relevant dimensions, the current study provides an initial attempt to evaluate effects of policy on public mental health.

There are several limitations to our study. Firstly, the data quality of health outcomes likely differs between countries and possibly within the same country over time. Since data providers were reluctant to send microdata, we requested aggregated data which, despite detailed specifications, might differ depending on who extracted the data for us. Secondly, we are unable to separate changes in true need or CMD rates triggered by the pandemic from confounding factors of changes in healthcare utilization (e.g., due to access and healthcare seeking behavior unrelated to need). Consequently, any conclusions about the effect of the pandemic and associated containment measures on public mental health based on care utilization data are limited. Thirdly, health effects of containment measures might be delayed. To evaluate which policy that had the largest impact, data from more countries allowing for random effect analyses would be needed. Finally, our analytical approach relies on comparisons of observed data relative to predicted data, which depends on the accuracy of our predictions. Although ARIMA models are a proven efficient analytical method for time series predictions, prediction accuracy would benefit from longer time series, both prior to the pandemic and during the pandemic, particularly when relating prevalence rates to containment measures.

### 4.3. Future directions

To circumvent limitations inherent in data of primary care utilization, future studies should combine different health outcomes to render a fuller picture of pandemic induced changes in public mental health. Furthermore, longer time series of healthcare utilization from more countries would allow for random effect analyses and more robust detections of associations between restriction policies and changes in mental health.

## 5. Conclusion

In conclusion, all countries but Latvia displayed a dramatic reduction in CMD prevalence in primary care during the first months of lockdowns. Whereas Norwegian and Dutch levels normalized by fall 2020, levels remained elevated in Latvia and low in Sweden through the year.

The sudden reductions in CMD prevalence in primary care indicate unmet needs. However, with the exception of Latvia, the current results do not confirm any pandemic related increase in CMD among the population in general. Neither could we convincingly confirm any associations between the studied containment measures and the mental health of the total population in general, or of school restrictions on children’s mental health in particular.

There are however clear age and sex dependent patterns of CMD change, where young people experienced large declines in CMD throughout 2020 in all countries, except in Sweden, where inverted age effects were observed. The differences in CMD care prevalence trajectories between countries and demographic groups are intriguing and warrants further investigation. Combining data on public mental health, such as prescription rates, population screenings of CMD and suicide rates would help differentiating the confounding effects of healthcare avoidance with changes in true need. This would be valuable when optimizing mental health interventions and resource allocations to meet the needs of the mostly affected population groups.

## Data availability statement

The raw data supporting the conclusions of this article will be made available by the authors, without undue reservation.

## Ethics statement

The studies involving human participants were reviewed and approved by the Swedish Ethical Review Authority, Uppsala, Sweden. Written informed consent from the participants’ legal guardian/next of kin was not required to participate in this study in accordance with the national legislation and the institutional requirements.

## Author contributions

PF: study design, conceptualization, data collection, data analysis, visualization, and preparation of the original draft. CD: conceptualization. AS, MK, KM, and LK: data collection. BT, PC, and EP: consulted for statistical analysis and access to containment measure indices. All authors contributed to the article and approved the submitted version.

## Footnotes

1
https://github.com/saudiwin/corona_index
