# Supplementary material for: Differential impact of the COVID-19 pandemic on primary care utilization related to common mental disorders in four European countries: A retrospective observational study
Source: Front Psychiatry. 2023 Jan 9;13:1045325. doi: 10.3389/fpsyt.2022.1045325 (PMC9868724; doi:10.3389/fpsyt.2022.1045325)
Supplement: Supplementary file 1 [file Table_1.docx]

Supplemental table 1. Pubmed search string used for identifying primary health care registers.

(

(

Hospital[tiab] OR Hospitals[tiab] OR Emergency[tiab] OR Surgery[tiab] OR Surgical[tiab] OR

Department[tiab] OR Departments[tiab] OR Unit[tiab] OR Units[tiab] OR Clinic[tiab] OR Clinics[tiab]

OR “Primary care”[tiab] OR Telemedicine[tiab] OR Telehealth[tiab]

)

AND

(

Admission[tiab] OR Admissions[tiab] OR Visit[tiab] OR Visits[tiab] OR Attendance[tiab] OR

Attending[tiab] OR Activity[tiab] OR Utilization[tiab] OR Utilisation[tiab]

)

)

AND

(

Psychiatric[tiab] OR Psychological[tiab] Mental[tiab] OR Depression[tiab] OR Depressive[tiab] OR Anxiety[tiab] OR GAD[tiab] OR PTSD[tiab]

OR Suicide[tiab] OR Suicidal[tiab] OR Schizophrenia[tiab] OR schizophrenic[tiab]

)

AND

(

"health records"[tiab] OR "health record"[tiab] OR "primary care"[tiab] OR "General Practitioner"[tiab] OR

"psychiatric hospital"[tiab] OR "patient records*"[tiab] OR "mental health service*"[tiab]

OR "register"[tiab] OR "patient visits"[tiab] OR "patient encounters"[tiab] OR recorded[tiab]

OR "database"[tiab] OR "mental health care"[tiab] OR "care utilization"[tiab] OR "admissions"[tiab]

OR "service utilisation"[tiab] OR "healthcare"[tiab] OR "number of contacts"[tiab] OR suicide*[tiab]

)

NOT

(

"health care worker*"[tiab] OR "healthcare worker*"[tiab] OR Nurses*[tiab]

OR "COVID-19 survivors"[tiab] OR "COVID-19 patients"[tiab] OR "survey"[tiab] OR "randomized-controlled"[tiab] OR "questionnaire"[tiab] OR "telephone interviews"[tiab]

)

AND

(

Russia*[tiab] OR Germany[tiab] OR German[tiab]OR "United Kingdom"[tiab] OR UK[tiab] OR British[tiab] OR

France[tiab] OR French[tiab] OR Italy[tiab] OR Italian[tiab] OR Spain[tiab] OR Spanish[tiab] OR

Ukraine[tiab] OR Ukrainian[tiab] OR Poland[tiab] OR Polish[tiab] OR Romania[tiab] OR Romanian[tiab] OR

Netherlands[tiab] OR Dutch[tiab] OR Belgium[tiab] OR Belgian[tiab] OR "Czech Republic"[tiab] OR czech[tiab] OR

Greece[tiab] OR Greek[tiab] OR Portugal[tiab] OR Portuguese[tiab] OR Sweden[tiab] OR Swedish[tiab] OR Hungary[tiab] OR Hungarian[tiab] OR

Belarus[tiab] OR Belarusian[tiab] OR Austria[tiab] OR Austrian[tiab] OR Serbia[tiab] OR Serbian[tiab] OR Switzerland[tiab] OR Swiss[tiab] OR

Bulgaria[tiab] OR Bulgarian[tiab] OR Denmark[tiab] OR Danish[tiab] OR Finland[tiab] OR Finnish[tiab] OR Slovakia[tiab] OR Slovakian[tiab] OR

Norway[tiab] OR Norwegian[tiab] OR Ireland[tiab] OR Irish[tiab] OR Croatia[tiab] OR Croatian[tiab] OR Moldova[tiab] OR Moldovan[tiab] OR

"Bosnia and Herzegovina"[tiab] OR Albania[tiab] OR Albanian[tiab] OR Lithuania[tiab] OR Lithuanian[tiab] OR

"North Macedonia"[tiab] OR Slovenia*[tiab] OR Slovenian[tiab] OR Latvia[tiab] OR Latvian[tiab] OR Estonia[tiab] OR Estonian[tiab] OR

Montenegro[tiab] OR Montenegrin[tiab] OR Luxembourg[tiab] OR Malta[tiab] OR Maltese[tiab] OR Iceland*[tiab]
