# Supplementary material for: Differential impact of the COVID-19 pandemic on primary care utilization related to common mental disorders in four European countries: A retrospective observational study
Source: Front Psychiatry. 2023 Jan 9;13:1045325. doi: 10.3389/fpsyt.2022.1045325 (PMC9868724; doi:10.3389/fpsyt.2022.1045325)
Supplement: Supplementary file 2 [file Table_2.docx]

Supplemental table 2. Disease classifications.

| Classification system | Anxiety disorders | Depressive disorders | All psychiatric conditions |
| --- | --- | --- | --- |
| ICD 10 | F40–45, F48 | F32-F33.9 | F* |
| ICPC2 | P74, P02, P 82, P79 | P76 | P* |
