# Supplementary material for: Differential impact of the COVID-19 pandemic on primary care utilization related to common mental disorders in four European countries: A retrospective observational study
Source: Front Psychiatry. 2023 Jan 9;13:1045325. doi: 10.3389/fpsyt.2022.1045325 (PMC9868724; doi:10.3389/fpsyt.2022.1045325)
Supplement: Supplementary file 3 [file Table_3.docx]

**Supplemental table 3.** *Non-linear relationship between containment measures and CMD prevalence* *in primary care.*

| Region | Measure | All ages, beta (p) | 0-14 yrs, beta (p) | 30-59 yrs, beta (p) | 75+ yrs, beta (p) |
| --- | --- | --- | --- | --- | --- |
| Sweden | SD | 1.3 x 10^-6 (.33) | 1.2 x 10^-7 (.57) | 1.3 x 10^-6 (.62) | 1.7 x 10^-6 (.12) |
|  | SR | 1.3 x 10^-6 (.80) | -1.2 x 10^-7 (.30) | 2.2 x 10^-6 (.90) | 3.6 x 10^-6 (.71) |
| Norway | SD | 3.6 x 10^-7 (.86) | 8.8 x 10^-8 (.39) | 1.9 x 10^-7 (.93) | 7.0 x 10^-7 (.35) |
|  | SD | 2.58 x 10^-7 (.91) | -6.5 x 10^-7 (.035)* | 1.2 x 10^-6 (.70) | -7.5 x 10^-7 (.64) |
| Netherlands | SD | -4.4 x 10^-9 (.96) | -1.5 x 10^-8 (.38) | -1.2 x 10^-9 (.99) | - 5.3 x 10^-8 (.18) |
|  | SR | -1.6 x 10^-7 (.15) | -9.4 x 10^-9 (.69) | -2.2 x 10^-7 (.14) | -5.1 x 10^-8 (.56) |
| Latvia | SD | -1.5 x 10^-7 (.34) | -1.2 x 10^-7 (.43) | -1.6 x 10^-7 (.29) | -1.2 x 10^-7 (.06) |
|  | SR | -6.8 x 10^-9(.84) | -8.1 x 10^-8 (.0020)* | 3.6 x 10^-8 (.45) | -1.9 x 10^-6 (.001)* |
