# Supplementary material for: Differential impact of the COVID-19 pandemic on primary care utilization related to common mental disorders in four European countries: A retrospective observational study
Source: Front Psychiatry. 2023 Jan 9;13:1045325. doi: 10.3389/fpsyt.2022.1045325 (PMC9868724; doi:10.3389/fpsyt.2022.1045325)
Supplement: Supplementary file 4 [file Image_1.pdf]

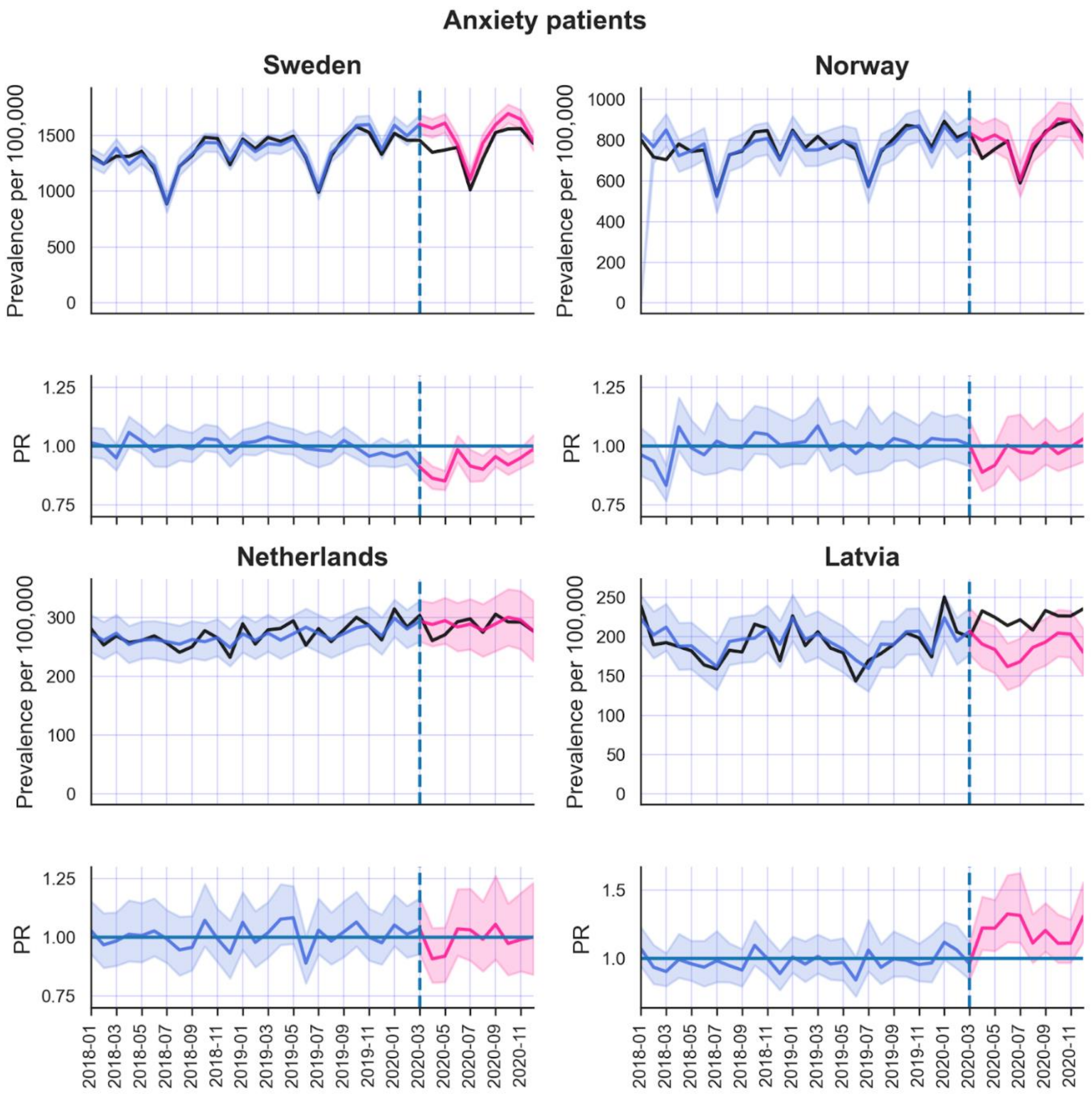

Supplemental figure 1. **Monthly counts of unique individuals with anxiety disorders.**

In 2019, the average monthly care prevalence in Sweden were 1400 individuals per 100 000 person-months, 780 per 100 000 person-months in Norway, 300 per 100 000 person-month in the Netherlands, and 200 per 100 000 person-month in Latvia.
