# Supplementary material for: Differential impact of the COVID-19 pandemic on primary care utilization related to common mental disorders in four European countries: A retrospective observational study
Source: Front Psychiatry. 2023 Jan 9;13:1045325. doi: 10.3389/fpsyt.2022.1045325 (PMC9868724; doi:10.3389/fpsyt.2022.1045325)
Supplement: Supplementary file 5 [file Image_2.pdf]

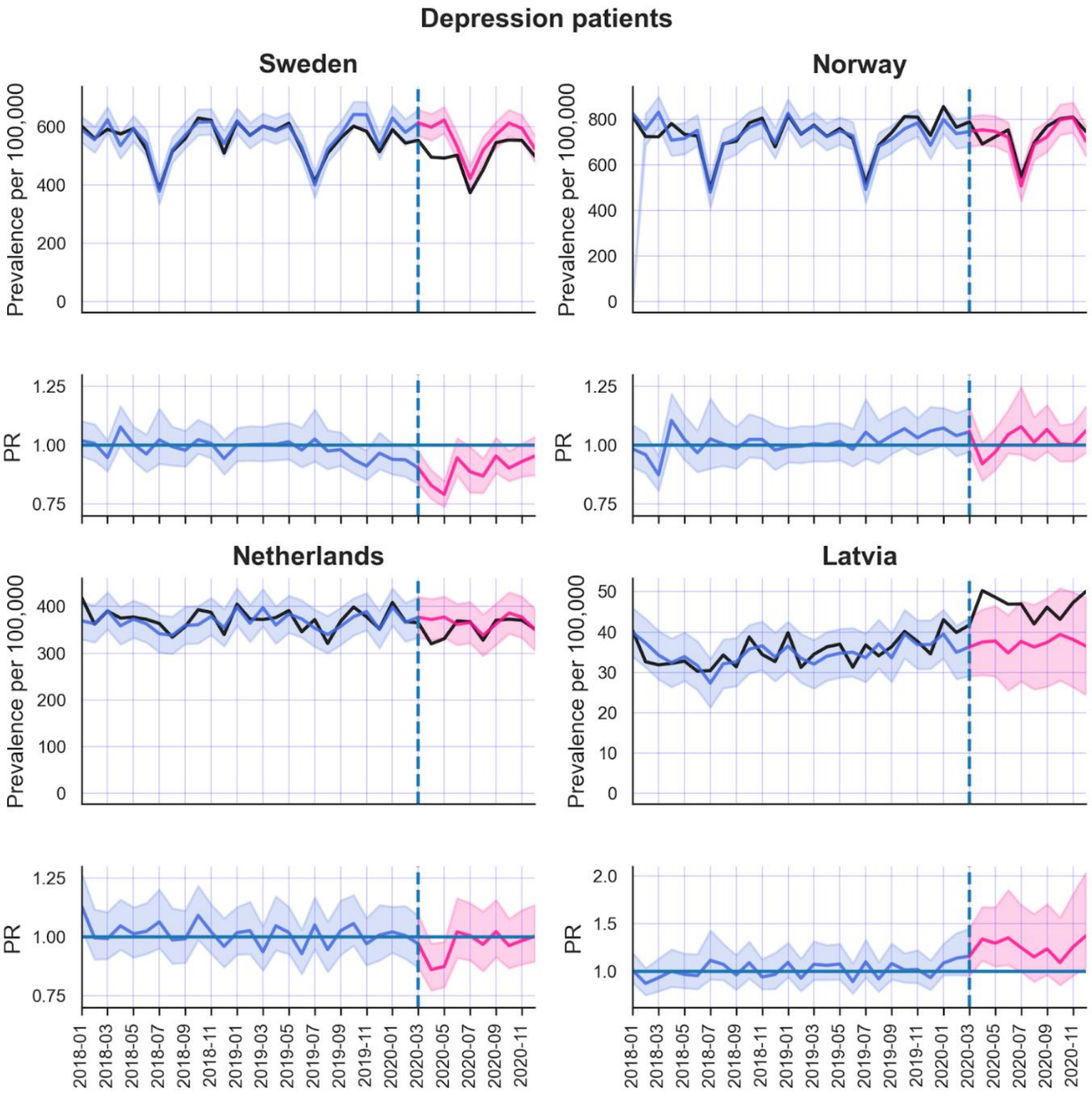

Supplemental figure 2. **Monthly counts of unique individuals with depressive disorders.**

Prevalence of depression prior to the pandemic varied from approximately 40 per 100 000 person-months in Latvia, to 750 per 100 000 person-months in Norway. The rates in Sweden and Netherlands were 500 and 350 per 100 000 person-months, respectively.
