# Supplementary material for: Differential impact of the COVID-19 pandemic on primary care utilization related to common mental disorders in four European countries: A retrospective observational study
Source: Front Psychiatry. 2023 Jan 9;13:1045325. doi: 10.3389/fpsyt.2022.1045325 (PMC9868724; doi:10.3389/fpsyt.2022.1045325)
Supplement: Supplementary file 6 [file Image_3.pdf]

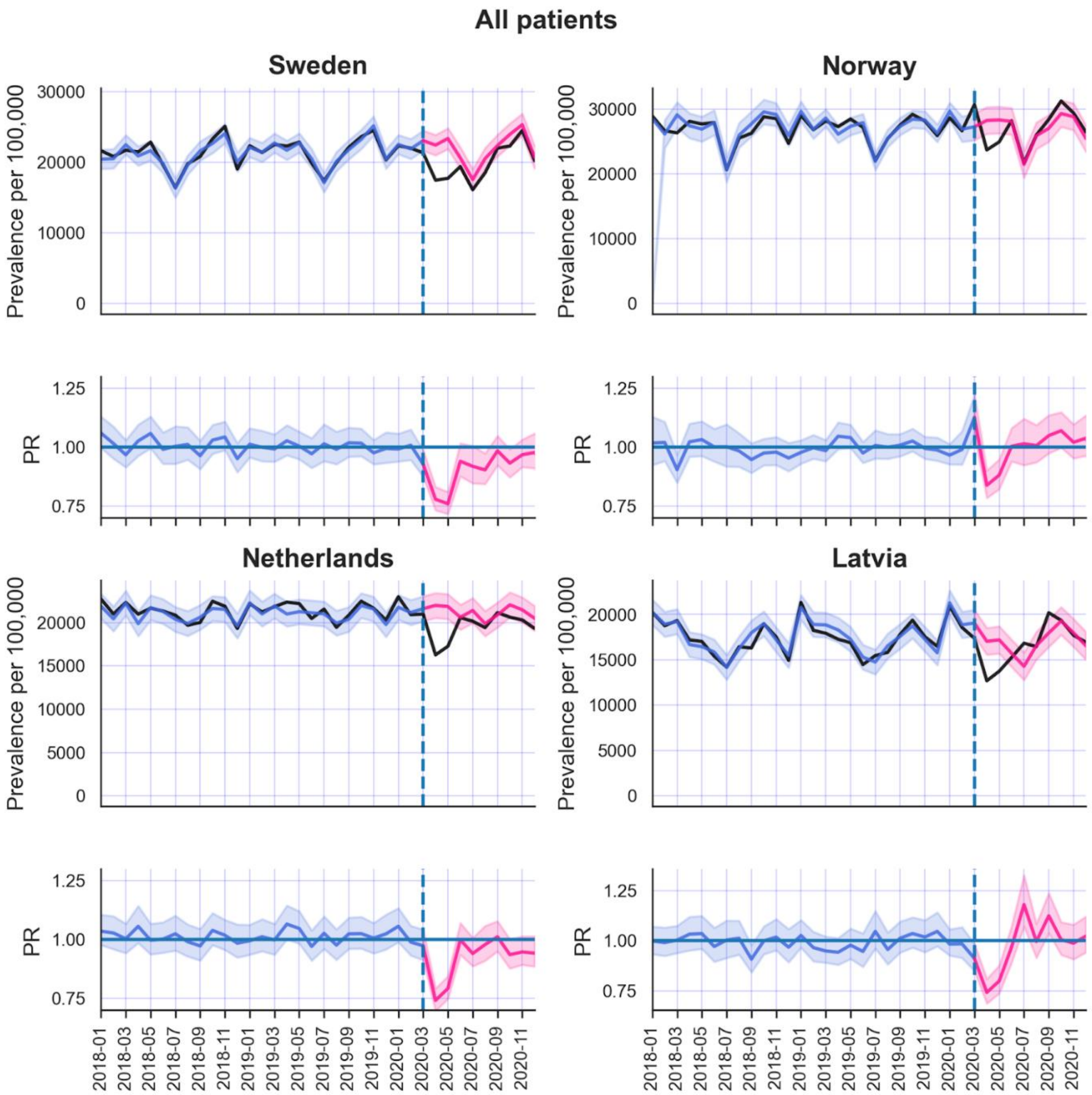

Supplemental figure 3. **Monthly counts of total number of unique patients in primary care.**

All four investigated countries displayed an reduction in care prevalence of any diagnosis in the early stage of the pandemic (mars 2020 to June 2020) of approxiamtly 15-25%, depending on country and months. Prior to the pandemic, monthly prevalence rates were lowest in Latvia (approx. 17500 cases per 100 000 person-months) per month and highest in Norway (27000 cases per 100 000), and roughly the same in Netherlands and Sweden (22000 cases per 100 000).
