# Supplementary material for: Differential impact of the COVID-19 pandemic on primary care utilization related to common mental disorders in four European countries: A retrospective observational study
Source: Front Psychiatry. 2023 Jan 9;13:1045325. doi: 10.3389/fpsyt.2022.1045325 (PMC9868724; doi:10.3389/fpsyt.2022.1045325)
Supplement: Supplementary file 7 [file Image_4.pdf]

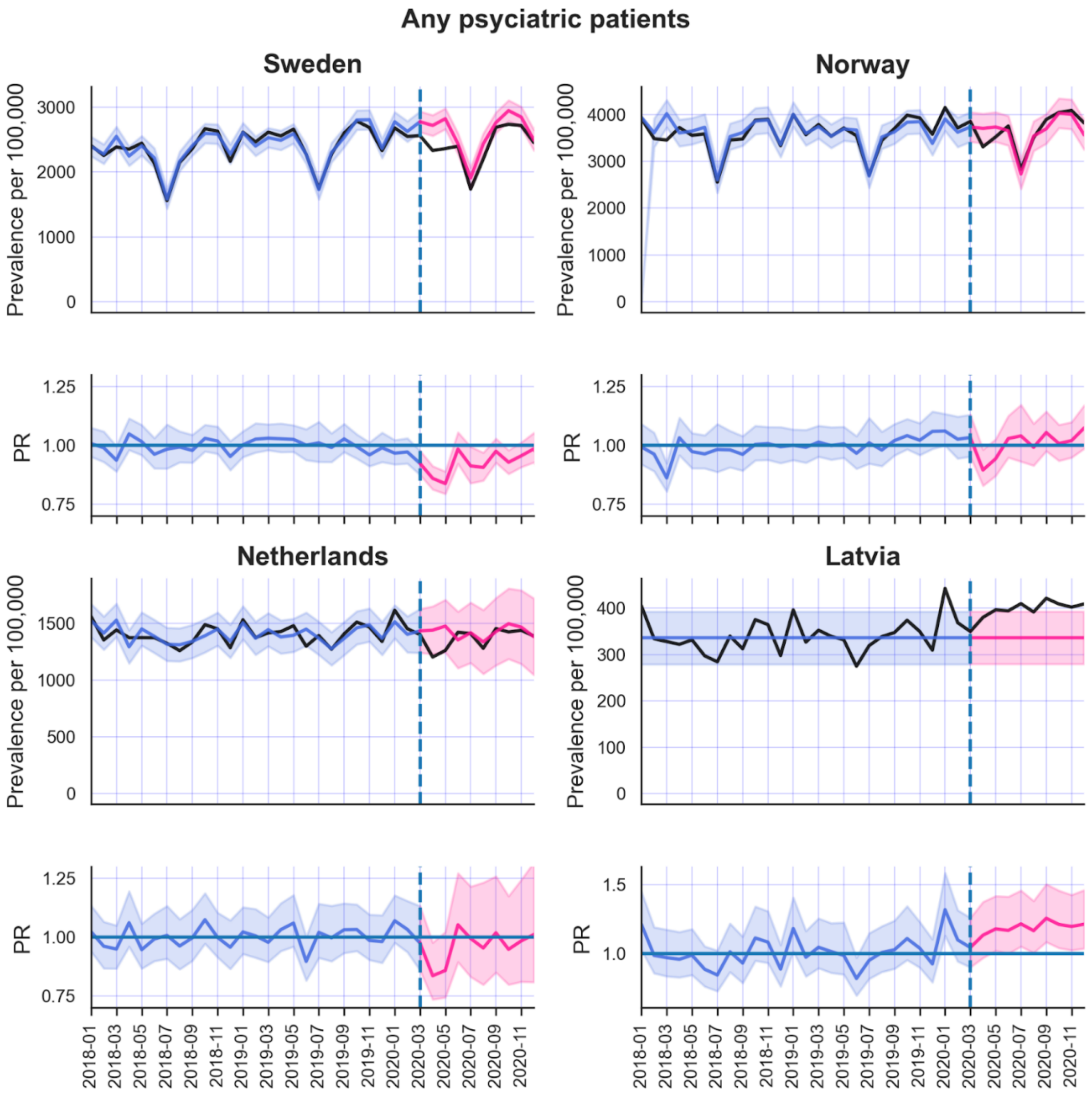

Supplemental figure 4. **Monthly counts of unique patients with any psychiatric diagnosis in primary care.**

Note, modeling of Latvian time series did not work well using SARIMA modeling for current time series, likely due to highly irregular and non seasonal data. .
