# Supplementary material for: Differential impact of the COVID-19 pandemic on primary care utilization related to common mental disorders in four European countries: A retrospective observational study
Source: Front Psychiatry. 2023 Jan 9;13:1045325. doi: 10.3389/fpsyt.2022.1045325 (PMC9868724; doi:10.3389/fpsyt.2022.1045325)
Supplement: Supplementary file 8 [file Image_5.pdf]

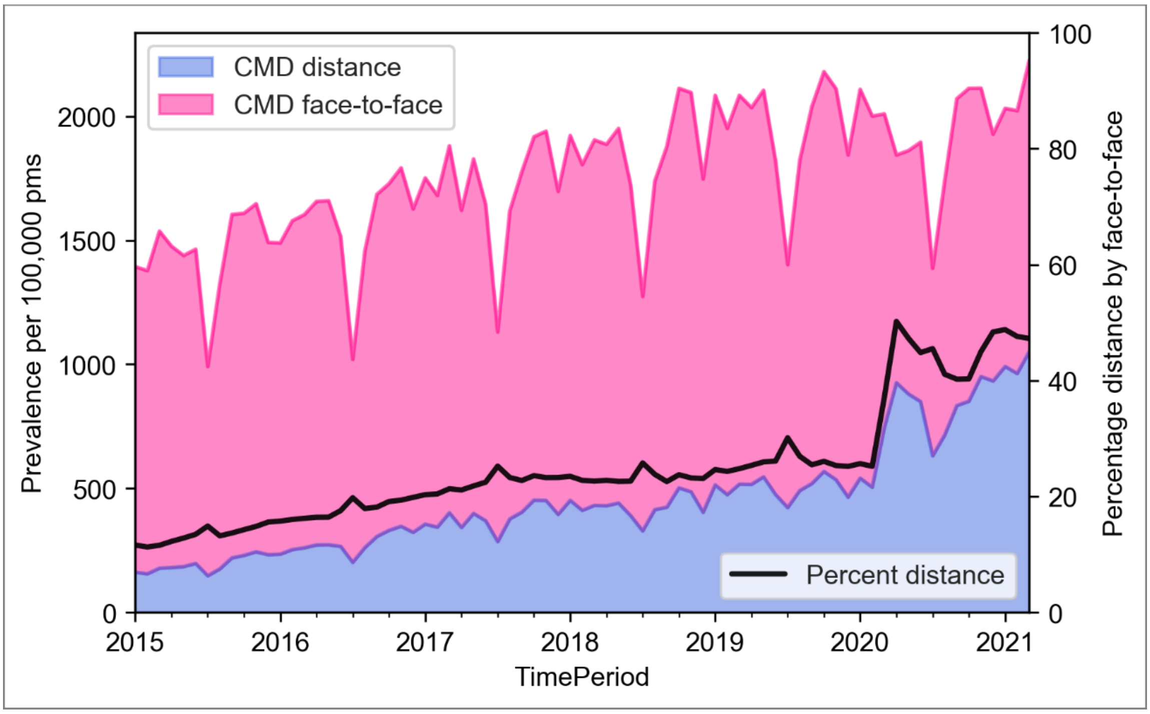

Supplemental figure 5. Changes in proportions of distance vs face-to-face CMD consultations in Sweden**.**

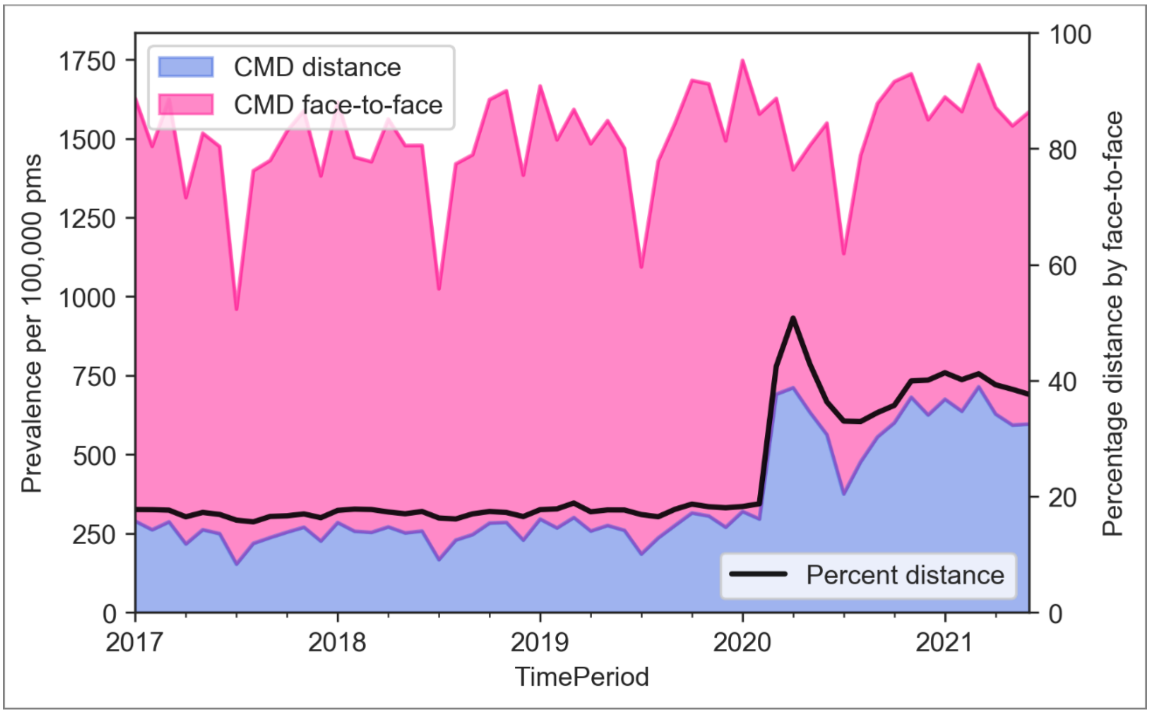

Supplemental figure 6. Changes in proportions of distance vs face-to-face CMD consultations in Norway.
